# Right-lateralized sleep spindles are associated with neutral over emotional bias in picture recognition: An overnight study

**DOI:** 10.3758/s13415-023-01113-4

**Published:** 2023-06-12

**Authors:** Risto Halonen, Sanni Luokkala, Liisa Kuula, Minea Antila, Anu-Katriina Pesonen

**Affiliations:** 1 SleepWell Research Program, Research Program Unit, Faculty of Medicine, University of Helsinki, P.O. Box 21, 00014 Helsinki, Finland; 2 Department of Psychology and Logopedics, Faculty of Medicine, University of Helsinki, P.O. Box 21, 00014 Helsinki, Finland

**Keywords:** Recognition memory, Lateralization, Sleep, Emotional memory, Sleep spindles, REM theta

## Abstract

Sleep is especially important for emotional memories, although the mechanisms for prioritizing emotional content are insufficiently known. As during waking, emotional processing during sleep may be hemispherically asymmetric; right-lateralized rapid-eye movement (REM) sleep theta (~4–7 Hz) is reportedly associated with emotional memory retention. No research exists on lateralized non-REM sleep oscillations. However, sleep spindles, especially when coupled with slow oscillations (SOs), facilitate off-line memory consolidation.

Our primary goal was to examine how the lateralization (right-to-left contrast) of REM theta, sleep spindles, and SO-spindle coupling is associated with overnight recognition memory in a task consisting of neutral and emotionally aversive pictures. Thirty-two healthy adults encoded 150 target pictures before overnight sleep. The recognition of target pictures among foils (discriminability, *d’*) was tested immediately, 12 hours, and 24 hours after encoding.

Recognition discriminability between targets and foils was similar for neutral and emotional pictures in immediate and 12-h retrievals. After 24 hours, emotional pictures were less accurately discriminated (*p* < 0.001). Emotional difference at 24-h retrieval was associated with right-to-left contrast in frontal fast spindle density (*p* < 0.001). The lateralization of SO-spindle coupling was associated with higher neutral versus emotional difference across all retrievals (*p* = 0.004).

Our findings contribute to a largely unstudied area in sleep-related memory research. Hemispheric asymmetry in non-REM sleep oscillations may contribute to how neutral versus emotional information is processed. This is presumably underlain by both mechanistic offline memory consolidation and a trait-like cognitive/affective bias that influences memory encoding and retrieval. Methodological choices and participants’ affective traits are likely involved.

**Supplementary Information:**

The online version contains supplementary material available at 10.3758/s13415-023-01113-4.

## Introduction

Memory consolidation during sleep is proposed to be selective in the sense that “salient” information is preserved (Kim & Payne, 2020). Affective features of a memory determine whether it is consolidated or forgotten; this is likely to promote survival under potentially threatening conditions. This postulates that sleep would preferentially consolidate emotional memories instead of neutral ones, and some influential behavioral studies do indeed align with the theoretical conceptualization (Hu et al., 2006; Payne et al., 2012). However, later systematic efforts to find an emotional enhancement specific to sleep, relative to wake, do not provide clear support for this view (Davidson et al., 2021; Lipinska et al., 2019; Schafer et al., 2020). Indeed, a closer examination of the sleeping brain dynamics is needed to elucidate specific sleep characteristics that facilitate the consolidation of emotional memories. Understudied characteristics are specifically related to hemispheric lateralization in both rapid eye movement (REM) sleep and non-REM (NREM).

Evidence from waking state studies indicates that emotional processing in the brain is asymmetric. For example, right-lateralized brain damage or atrophy may lead to deficits in both perception and comprehension of emotional material (Ahern et al., 1991; DeKosky et al., 1980; Gainotti, 2019; Rosen et al., 2002), as well as to hypoarousal in response to arousing stimuli (Glascher & Adolphs, 2003). Also noninjured brains show lateralized emotion processing. Functional asymmetry has been observed to mirror trait-like response tendency toward emotional stimuli, such that increased right-hemisphere activity associates with negative emotionality (Balconi et al., 2017; Balconi & Mazza, 2010). In a closer look at clinical populations, depression and comorbid anxiety are linked to lower left frontal cortical activity than right frontal cortical activity (Bruder et al., 1997; Bruder et al., 2017).

How does asymmetric brain activity relate to emotional memory processing during sleep? Especially REM sleep is considered a sleep stage for emotional processing (Genzel et al., 2015; Hutchison & Rathore, 2015; Rasch & Born, 2013; Walker, 2009; Walker & van der Helm, 2009). Accordingly, behavioral studies indicate that REM sleep and its major oscillatory correlate for memory processing, the prefrontal theta (4-7 Hz), play an important function in emotional memory consolidation (Nishida et al., 2009; Sopp et al., 2017). Remarkably, the relative hemispheric difference in prefrontal theta, that is, lateralization, appears consequential; elevated right-lateralized theta associates with the retention of especially emotional material (Nishida et al., 2009; Sopp et al., 2017).

The role of NREM sleep in emotional memory is less known. Sleep spindles and their synchronized occurrence with slow oscillations (SOs) can facilitate memory consolidation during sleep (Klinzing et al., 2019; Ngo et al., 2015; Rosanova & Ulrich, 2005), which is supported by consistent behavioral evidence (Clemens et al., 2005; Goder et al., 2015; Halonen et al., 2019; Halonen et al., 2021a; Mikutta et al., 2019; Muehlroth et al., 2019). Interestingly, some studies propose that (SO-coupled) sleep spindles may be associated more strongly with the retention of emotional, relative to neutral, material (Cairney et al., 2014; Denis et al., 2022; Halonen et al., 2022; Kaestner et al., 2013). However, it remains unknown whether the lateralization of these oscillations is relevant. One study linked frontal spindles on the right hemisphere with better memory performance for emotional—but not neutral—material (Cairney et al., 2014), whereas another study reported a similar benefit for neutral associative memory (Sopp et al., 2018). Whereas these reports hint at a mechanism relating emotional memory processes to lateralized oscillations, the hemispheric asymmetry of NREM events in emotional offline memory consolidation remains unstudied. Of note, spindle activity and SO-spindle coupling strength are modulated in a local manner based on presleep–waking activity (Morin et al., 2008; Yordanova, Kirov, et al., 2017a; Yordanova, Kolev, et al., 2017b). Given the accentuated role of the right hemisphere in affective processing, it is important to study whether NREM oscillations mirror such lateralization.

In summary, previous research indicates that characteristic parameters of both NREM and REM sleep relate to the consolidation of emotional memories. Some studies have linked frontal theta activity to emotional memory performance in a lateralized manner, right prefrontal theta predicting enhanced memory performance for emotional material. This finding suggests that the known importance of the right hemisphere in emotion processing extends to sleep-related memory consolidation. Whether oscillatory lateralization also applies to sleep spindles and SO-spindle coupling activity is not yet known. Thus, the purpose of this study was to investigate how the lateralization of oscillatory characteristics in both REM and NREM sleep (i.e., REM theta, sleep spindles, and SO-spindle coupling) predicts overnight memory performance. Of specific interest was to examine whether the affective strength of the memorized material (neutral or aversive pictures) interacts with oscillatory parameters and their lateralization. Our hypothesis was that right-lateralized prefrontal REM theta is associated with elevated performance in emotional, rather than neutral, picture recognition. We also expected that right-lateralized spindle density is associated with similar memory bias for emotional items, whereas nonlateralized (SO-coupled) spindle activity would positively predict memory retention overall.

## Methods

### Participants

The initial sample consisted of 34 young adults (26 females) living in the capital area of Finland. Different sources were used to recruit participants; 11 participants were invited from the previously studied *SleepHelsinki!* cohort (see details of the cohort1), 15 were students at the University of Helsinki who were contacted via e-mail lists and social media platforms within student societies, and three were invited through personal contacts. For their participation, a monetary compensation of 100€ was provided to all participants. Measurements were performed between June 2020 and January 2021. The participants also answered questionnaires for handedness, depressive symptoms [Beck Depression Inventory (BDI)] (Beck et al., 1996), and generalized anxiety symptoms [Generalized Anxiety Disorder-7 (GAD-7)] (Williams, 2014) in order for this symptomology to be addressed in analyses evaluating memory function. The participants did not have any diagnosed sleeping disorders, nor did they use medication known to affect sleep. Data from two participants were discarded due to technical problems in polysomnography recording.

All participants provided written, informed consents before study participation. The study protocol was approved by the Helsinki University Hospital Ethics Committee (HUS/1390/2019), and all components of the study were conducted in accordance with the Declaration of Helsinki and its later amendments.

### Study design

**Fig. 1:**
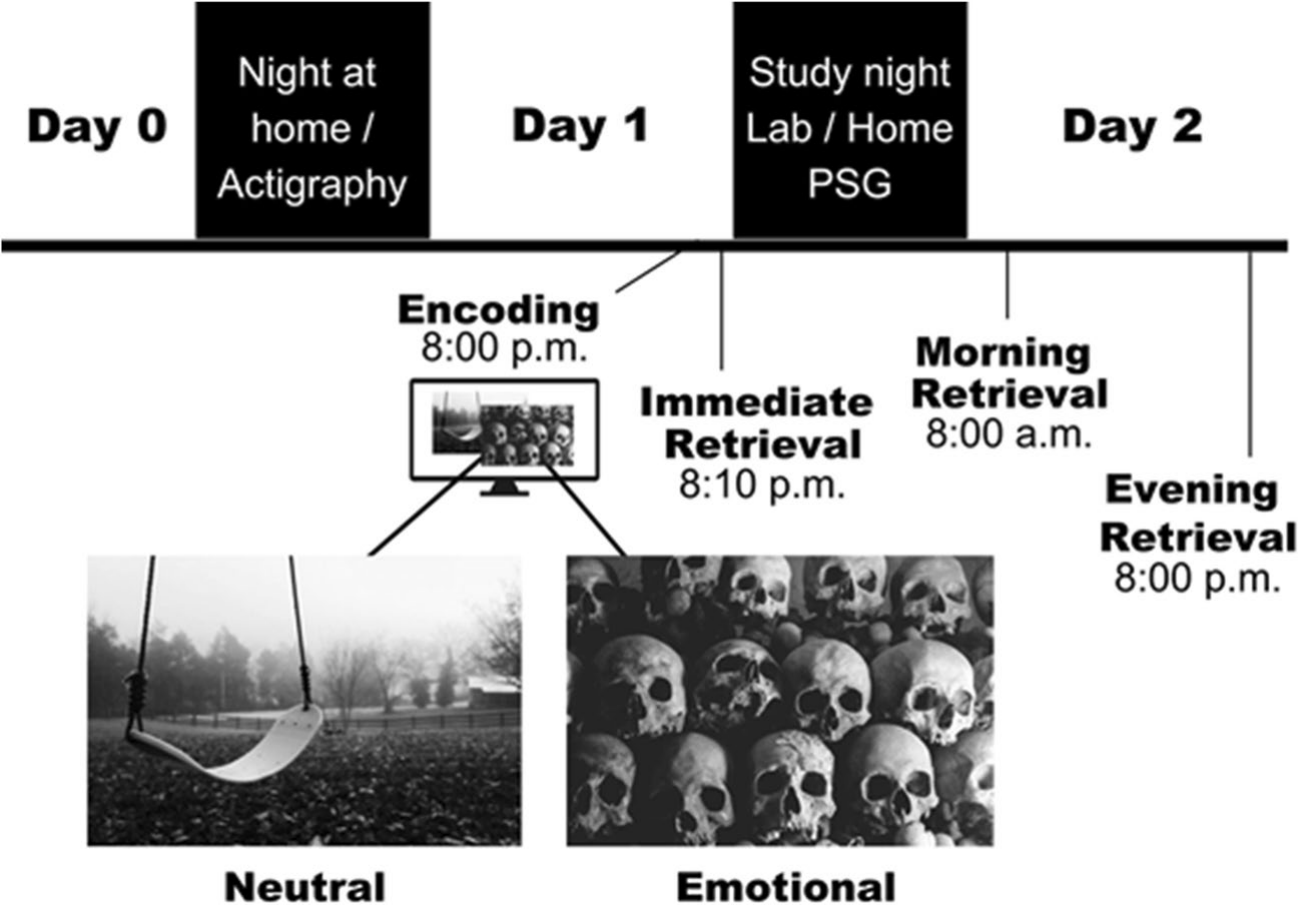
After encoding of emotional and neutral picture stimuli, the study design included three retrieval occasions (immediate, morning, and evening). During the study night, the participants underwent PSG measurement procedure

After enrollment in the study, background data were collected from participants via electronic questionnaires (BDI, GAD-7, learning impairments and health status). On the night between Days 0 and 1, all participants slept at home and sleep was measured with actigraphy. To minimize possible hesitancy to take part in the study during the ongoing COVID-19 pandemic, we let the participants choose the location of the study night (i.e., the night between Days 1 and 2), either in the sleep laboratory or at the participant’s home. Fifteen participants (47%) chose the laboratory premises. On the evening of Day 1 (~8 pm), the research assistant met the participants at home or at the sleep laboratory. After this, the picture encoding took place followed by an immediate retrieval. After a polysomnography (PSG) device was attached, the participants had the opportunity to sleep 11 pm and 07 am. The first delayed retrieval (i.e., 12-h) took place the next morning (~8 am). The participants spent Day 2 of the study engaged in their normal activities. The second delayed retrieval (i.e., 24-h) took place in the evening (~8 pm) on Day 2. Figure 1 illustrates the study design.

## Material and memory task

The computerized recognition memory task comprised pictures selected from the International Affective Picture System (IAPS), a picture set with standardized emotional ratings that for valence vary from negative to positive (1-9) and for arousal from calm to exciting (1-9) (Lang et al., 2005). The stimuli consisted of altogether 300 pictures, of which 150 were neutral (*M* = 5.47 for valence, *M* = 3.25 for arousal) and 150 negatively emotional (*M* = 2.99 for valence, *M* = 5.45 for arousal). Negative and neutral pictures differed significantly in both valence (*F* = 1082.5, one-way analysis of variance *p* < 0.001) and arousal (*F* = 9.533, *p* < 0.001). Between immediate, 12-h, and 24-h conditions, the stimuli were balanced for valence (*p* = 0.991) and arousal (*p* = 0.965). Likewise, the stimuli presented on different retrieval occasions were balanced between target items and new foils for valence (*p* = 0.759) and arousal (*p* = 0.931). In the encoding, participants were instructed to memorize all displayed pictures. Each picture was displayed for 2,000 ms on a 14” screen followed by a 1,000-ms interval during which a fixation cross was displayed in the middle of the black screen. The participants encoded 150 pictures, first a set of 75 neutral pictures in random order, followed immediately by a set of 75 negative pictures in random order. This sequential presentation prevented evoking affective arousal during the encoding of neutral pictures, but only during (the latter set) of aversive material.

On each retrieval occasion (immediate, 12-h, and 24-h), the participants were shown 50 target pictures, intermixed with 50 new pictures (foils) not previously seen. All sets of target pictures and new foils included 25 neutral and 25 emotional pictures. In each trial, the subjects classified the pictures either as recollected, familiar, or new by pressing a button numbered either 1 (recollection), 2 (familiar), or 0 (new). The participants were instructed to choose option 1 if they recalled encoding the displayed picture and option 2 if the displayed picture provided a sense of familiarity without specific recall of the encoding moment. From these answers, total hit rate was calculated for items classified correctly as recollected or familiar. Total false-alarm hit rate was calculated for items incorrectly classified as recollected or familiar. After this, an index of recognition discriminability (i.e., the ability to distinguish the target pictures from foils, *d’*) was calculated separately for emotional and neutral items as a difference between z-transformed probabilities of hit and false-alarm rates: *d’* = z (hit rate) – z(false-alarm hit rate). Thus, six *d’* scores were obtained (3 retrievals, 2 emotion categories). Recollection-based *d’* (only considering “recollection” answers) was examined in Supplementary material 1.

### Actigraphy

On Day 0, the participants were given Philips Actiwatch 2 actigraphs. On the night between Day 0 and Day 1, sleep was measured using the actigraphy devices to screen for highly deviant sleep durations.

### Polysomnography and preprocessing

All recordings were performed by using either SOMNOscreen plus or SOMNOscreen HD (SOMNOmedics GmbH, Randersacker, Germany). Gold cup electrodes were attached at six EEG locations [frontal (F) hemispheres F3 and F4; central (C) C3 and C4; occipital (O) O1 and O2; mastoid (A1, A2)] by a trained research nurse. Disposable adhesive electrodes (Ambu Neuroline 715; Ambu A/S, Ballerup, Denmark) were used to measure electrooculograms (EOGs) and chin electromyograms (EMGs), with two electrode locations for EOG and three locations for EMG. Additionally, an online reference (Cz) and a ground electrode were attached. The sampling rate was set at 256 Hz (the hardware filters for SOMNOscreen plus were 0.2–35 Hz). The DOMINO program (v2.7; SOMNOmedics GmbH, Germany) was used to score the PSG data manually in 30-s epochs into N1, N2, N3 (SWS), REM, and wake according to AASM guidelines (AASM Manual for the Scoring of Sleep and Associated Events, 2007). Additionally, in the epochs scored as sleep, we visually identified and marked artifact segments, that is, pronounced EEG potential bursts caused by an extracerebral source, e.g., muscle activity, body movements, and short arousals (Kane et al., 2017).

The manually scored PSG signals were converted to EDF format in DOMINO software (SOMNOmedics GmbH, Germany) and then further analyzed using the functions of EEGlab 14.1.2b (Delorme & Makeig, 2004) running on MATLAB R2018a (MathWorks, Inc., Natick, MA). All signals were digitally band-passed and filtered offline from 0.2 to 35 Hz (with a Hamming windowed sinc zero-phase FIR filter; cutoff, −6 dB), at 0.1 and 35.1 Hz, respectively, and re-referenced to the average signal of A1 and A2 electrodes. Electrodes located at F3, F4, C3, and C4 were included in further analyses. Pre-scored epochs with major (>8 s) artifacts or contact impedance higher than 30 kOhm in the target electrode were excluded from further analyses.

### Sleep spindle detection

The preprocessed EEG data were further band-pass filtered (order 2816) into fast spindle (13–16-Hz) frequency band. Spindles were extracted from the filtered signal using a method based on an automated detection algorithm described by Ferrarelli et al. (2007). For finding the spindle peak amplitude in each channel, the threshold values were defined by the mean of the channel amplitude (μV) multiplied by 5. Resulting in minimum spindle duration of 0.5 s, the putative spindle’s amplitude was required to stay over the mean channel amplitude multiplied by 2 for 250 ms in both directions from the peak maximum. Considering the possible signal variation across channels and between individuals, we used channel-wise threshold definitions. The maximum cutoff for spindle length was set to 3.0 s, and the maximum peak amplitude was set to 200 μV. Additionally, the signal amplitude between spindles was required to stay under the lower threshold for 78.1 ms, which is approximately the duration of one period of sine at 13 Hz. This requirement was implemented to prevent false alarms. Finally, spindle-like bursts that occurred during any segments marked as artifacts were excluded. The detection was run for NREM sleep (combining stages N2 and N3) epochs. Sleep spindle density (number/minute) was determined separately for each electrode.

We averaged the spindle densities from frontal (F3, F4) and central (C3, C4) electrodes (i.e., frontal spindle density and central spindle density). Additionally, we created Spindle^Lat^ variable for frontal derivation to represent the relative right-versus-left regions’ activity difference by subtracting the fast spindle density (13-16 Hz) at F3 from the fast spindle density at F4, and divided by their mean.

### SO-spindle coupling distance

Slow oscillations were detected with an adapted algorithm developed by Ngo et al. (2015) using the Wonambi EEG analysis toolbox (Piantoni & O’Byrne, 2022; Wonambi: EEG analysis toolbox v.6.13). The signal was first low-pass filtered at 3.5 Hz. All peaks for negative and positive amplitudes were identified between consecutive positive-to-negative zero-crossings, comprising a full phase cycle. Within the duration of 0.8–5 s, zero-crossing intervals were included, corresponding to the 0.2–1.25 frequency range. Finally, mean values were calculated for positive and negative peak potentials, and these events were denoted as SOs, where the negative peak was lower than the mean negative peak and where the positive-to-negative peak amplitude difference exceeded the mean amplitude difference. The detection procedure was run for NREM (N2 + N3) sleep.

For NREM (N2 + N3) sleep, we calculated the channel-wise coupling distance and mean phase for SO-coupled fast spindles. First, we identified spindles where the amplitude peaked within an SO cycle (i.e., SO–spindles). Next, we band-pass filtered the EEG signal to 0.2–1.25 Hz, Hilbert-transformed the SO signal, and extracted the instantaneous phase at the SO–spindle peaks. Coupling distance, i.e., the phase difference in radians (absolute values) between the spindle peak and the positive SO-peak (0°), as well as the mean coupling phase were obtained for each SO-spindle with CircStat toolbox (Berens, 2009).

We averaged the channel-wise mean coupling distances in frontal (F3, F4) and central (C3, C4) electrodes (i.e., variables frontal and central mean coupling distance). Additionally, the relative right-versus-left difference in mean coupling distance, Coupling^Lat^, was created for frontal regions by subtracting the SO-spindle coupling distance in the left hemisphere from the value in the right hemisphere and dividing by their mean.

### Spectral power density analysis

The preprocessed signals were epoched with a 30-s window length. For the epochs scored as REM sleep, the “spectopo” function of EEGlab (1024 samples and overlap of 50%) was used to calculate power spectral density (PSD) for delta (0.5–4 Hz), theta (4–7 Hz), alpha (8–12 Hz), sigma (12–16 Hz), beta1 (16–22 Hz), and beta2 (22–30 Hz) bands in F3 and F4 channels. Channel-wise PSD in theta band was then standardized across all frequency bands and both frontal channels. This was done in order to prevent inter-individual variation in overall PSG amplitude (due to skull/skin properties or electrode conductance) from biasing the PSD values and to enable observation of the relative strength of specifically theta band in the full spectrum, while maintaining the difference between F3 and F4 values. We averaged the theta PSD from the frontal electrodes (i.e., REM Theta). The relative right-versus-left difference in frontal theta power density, Theta^Lat^, was created by subtracting the activity at F3 from the activity at F4 and dividing by their mean.

### Statistical analyses

We used IBM SPSS Statistics 29 in all analyses. The distributions of the analyzed variables were investigated for possible outliers and skewness. To investigate the suitability of linear models as the analytic tool of choice, residual scatter plots were created and then visually investigated to exclude the possibility of heteroscedasticity of residual distribution. Also, multicollinearity between the dependent variables—Spindle^Lat^, Coupling^Lat^, and Theta^Lat^—and the covariates was investigated.

We ran a set of preliminary analyses for identifying potential confounders. One-way analysis of variance (ANOVA) was used to test whether age, sleep, and oscillatory characteristics and questionnaire scores differed according to sex or the location of measurement. Chi-squared test was applied to test whether the source of recruitment (*SleepHelsinki!* cohort vs. other sources) affected the location of measurement (home/laboratory). Pearson’s correlation was used to examine possible associations between BDI and GAD-7 scores and sleep and oscillatory characteristics, as well as the recognition discriminability (*d’*) scores. Due to right-skewed BDI and GAD-7 distributions, these variables were log-transformed (natural logarithm). One participant lacked BDI and GAD-7 scores, and the missing values were replaced with sample means. We also tested age and sleep characteristics for significant correlations with *d’*.

To examine the recognition discriminability (*d’*) scores across the three retrievals and over two emotion categories, we used a two-way repeated measures ANOVA. In the analysis, *d’* was set as the dependent variable with two levels (neutral/emotional). The timing of the memory retrieval task was set as the independent variable with three levels (immediate/12-h/24-h). Multiple paired *t*-tests were run as post-hoc tests to reveal the exact time points between which *d’* differed, and at which time points *d’* measures for emotional and neutral items differed from each other. Bonferroni correction was used to keep the Type I error at 5% level overall. A similar exploratory analysis was conducted for hit rates and false alarm rates as dependent variables instead of *d’*.

The effect of oscillatory/lateralization variables (i.e. spindle density, coupling distance, and REM Theta / Spindle^Lat^, Coupling^Lat^, and Theta^Lat^) on recognition discriminability (*d’*) across all three retrievals were tested with repeated measures ANCOVA by assigning an oscillatory variable (each separately) as a continuous independent variable in the model described above. Thus, we examined the oscillatory/lateralization variables for main effect (i.e., the association with *overall d’*, across all retrievals) and for interaction with time (i.e. the association with *d’ change* between retrievals), emotion (i.e. the association with the *difference* between neutral and emotional picture *d’*), and “time x emotion” (i.e., the association with the change of emotional difference between the three retrievals). In case of significant interactions, we followed up by calculating respective difference scores (time: immediate *d’* – 12-h *d’* and 12-h *d’* – 24-h *d’*; emotion: neutral *d’* – emotional *d’*; time x emotion: neutral *d’* – emotional *d’* in each retrieval) and tested their association with oscillatory/lateralization variable with linear regression. As exploratory, we conducted the follow-up analyses for hit rates and false alarm rates as dependent variables.

In all repeated-measures, ANCOVA analyses regarding oscillatory/lateralization variables and picture retrieval, we controlled for sex, measurement location, and sleep duration (Model 1). In addition, the analyses were run in a model where also BDI and GAD-7 were controlled (Model 2). In the follow-up regression tests, we applied the same covariates as in the significant interactions. Additionally, when examining difference scores from a later retrieval (e.g., 24-h), we also controlled for the similar difference score from the previous retrieval (e.g., 12-h). Hence, we could isolate the change that took place between the retrievals. The impact of handedness on significant lateralization variables was examined by assigning it as an additional covariate in the follow-up analyses. We opted not to use it as a persistent covariate due to four missing values, instead imputing a distinct categorical value in these cases.

As a supplementary exploration (Supplementary material 3), we examined how subgroups formed by a median split on either Coupling^Lat^ or Spindle^Lat^ differed in terms of *d’* across the three measurements. One-way ANOVA was used to test for subgroup differences in age and sleep/oscillatory measures. A mixed ANCOVA was created by assigning the subgroup status as the between-subjects variable in the model described previously (*d’* with 2 emotions and 3 retrievals as dependent variable; covariates as in Model 1).

No *a priori* power calculations were conducted. The sample size was determined on the basis of previous studies examining sleep physiology and memory, conducting experiments with ~20–40 participants (Cairney et al., 2014; Mikutta et al., 2019; Muehlroth et al., 2019; Sopp et al., 2017). Sensitivity analyses applied with G*Power (Faul et al., 2007) showed that a sample of 32 could detect medium-to-large effect sizes with 80% power and alpha level of 0.05 in the types of analyses conducted in this study.

## Results

### Sample characteristics

Of the 32 participants (25 females), 26 were right-hand dominant, one was left-hand dominant, and one was ambidextrous. Data on handedness were lacking for four participants. Examining the variables’ distributions revealed that frontal and central spindle densities in one participant exceeded the means by more than 3 SD. These values were excluded from further analyses. Table 1 presents sample characteristics, including age, sleep measures, and questionnaire scores.

**Table 1:** Sample characteristics (N = 32)

			Range	Mean	SD	Home vs. laboratory *p*
Age			18.98–40.90	21.93	4.02	0.103
Sleep characteristics						
	Sleep duration, previous night (h:mm)		5:50–9:18	7:14	0:50	0.136
	Sleep duration, study night (h:mm)		4:18–7:49	6:44	0:45	0.010*
		N1 %	1.44–17.26	5.74	3.37	0.048*
		N2 %	21.63–49.89	36.32	8.31	0.951
		N3 %	8.15–41.44	24.04	7.38	0.066
		REM %	7.87–27.91	18.39	5.08	0.191
	WASO (h:mm)		0:07–3:09	0:43	0:46	0.776
Oscillatory characteristics						
	Fast spindle density F		2.23–5.01	3.33	0.66	0.215
	Fast spindle density C		2.66–5.03	3.79	0.57	0.175
	SO amount F / channel		548–1322	977	201	0.007**
	SO amount C / channel		510–1215	929	182	0.028*
	Coupling distance F (rad)		0.49–1.29	0.95	0.19	0.505
	Coupling distance C (rad)		0.59–1.18	0.88	0.15	0.839
BDI score GAD-7 score			0–45	11.75	10.85	0.517
			1–16	5.66	4.33	0.622

*SD* standard deviation, *N1-3* nonrapid eye movement sleep stages 1-3, *REM* rapid eye movement sleep, *WASO* wake after sleep onset, *SO* slow oscillation, *F* frontal, *C* central, *BDI* Beck Depression Inventory, *GAD-7* General Anxiety Disorder-7. **p < 0.01; *p < 0.05.

In our preliminary inspection, we found that females had significantly higher central spindle density (*p* = 0.023) and lower frontal coupling distance (*p* = 0.042) than males. Sleep duration correlated positively with emotional *d’* in immediate and 24-h retrieval and neutral *d’* in 12-h retrieval. Those participants who spent the study night at home had longer sleep duration (*p* = 0.010) and more SOs in frontal (*p* = 0.007) and central electrodes (*p* = 0.028), as well as lower N1 % (*p* = 0.048) than those who slept in the laboratory (Table 1). Additionally, all *SleepHelsinki!* participants chose home measurement, whereas 83% of those recruited via other sources slept in the laboratory (chi-squared *p* < 0.001). Thus, we assigned sex, sleep duration, and measurement location as covariates in all analyses between oscillatory/lateralization variables and discriminability *d’* (Model 1).

Analyses between questionnaire scores and sleep measures revealed that GAD-7 score correlated significantly with BDI (r = 0.800, *p* < 0.001), central spindle density (r = −0.430, *p* = 0.016), and Spindle^Lat^ (*r* = −0.490, *p* = 0.004), meaning lower GAD-7 scores in those with relative right-emphasized spindle density. BDI correlated negatively with WASO (r = −0.402, *p* = 0.023). Regarding discriminability *d’*, GAD-7 correlated with neutral *d’* in immediate retrieval (r = −0.380, *p* = 0.032) and both neutral (r = −0.552, p = 0.001) and emotional (r = −0.440, p = 0.012) *d’* at 24-h retrieval. BDI correlated with neutral *d’* in the 24-h retrieval (r = −0.399, *p* = 0.024). These associations prompted us to use GAD-7 and BDI scores as covariates in Model 2.

### Picture recognition across retrievals

**Fig. 2:**
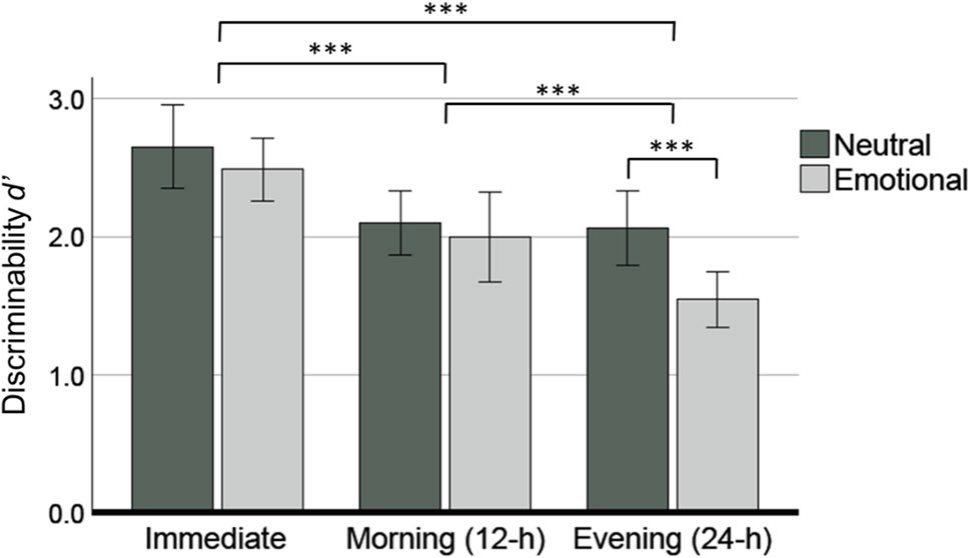
Discriminability *d’* for neutral and emotional pictures across immediate, 12-h and 24-h retrievals. *p < 0.05; **p < 0.01; ***p < 0.001. Error bars indicate 95% confidence interval

The discriminability between target and foil pictures (*d’*) differed significantly between different time points of memory retrieval [*F*(2, 62)= 47.887, *p* < 0.001]. Also, emotion had a significant main effect in the model [*F*(1, 31) = 10.459, *p* = 0.003]. Finally, time and emotion had a significant interaction in the model [*F*(2, 62) = 4.496, *p* = 0.015]. Post-hoc analyses conducted using Bonferroni-corrected pairwise comparisons revealed that *d’* decreased from immediate retrieval (*M* = 2.57, *SE* = 0.11) to morning retrieval (*M* = 2.05, *SE* = 0.13) significantly (*p* < 0.001). Also, the decrease in *d’* from immediate to evening retrieval (*M* = 1.81, *SE* = 0.11) was significant (*p* < 0.001). Likewise, the decrease in *d’* from morning retrieval to evening retrieval was significant (*p* = 0.010). Additionally, *d’* for neutral items at evening retrieval (*M* = 2.06, *SE* = 0.13) was significantly (*p* < 0.001) greater than *d’* for emotional items (*M* = 1.55, *SE* = 0.1). Figure 2 displays the results of the two-way repeated measures ANOVA described above. See Supplementary material 1 for recollection-based *d’*.

### Sleep oscillations and recognition discriminability

**Fig. 3:**
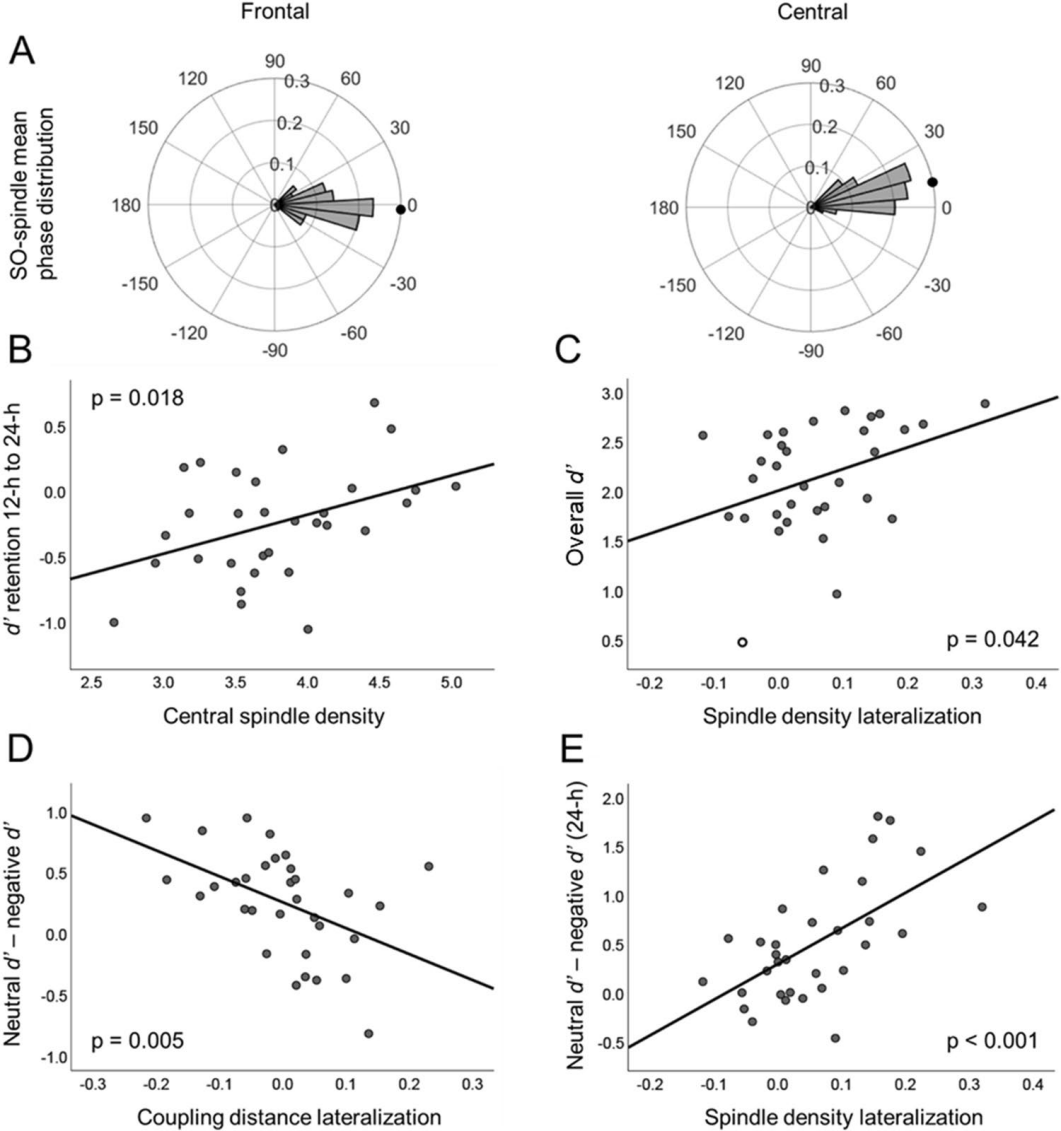
Sleep oscillations and discriminability *d’*. All scatterplots are drawn according to Model 1. (**A**) Polar histograms representing the nonuniformly distributed (*p* < 0.001) mean phases of SO-coupled frontal and central sleep spindles. The grand mean phases for frontal and central SO-spindles were −2.3° and 12.9°, respectively (dots). (**B**) Central spindle density was significantly associated with retention performance between 12-h and 24-h retrievals (*p* = 0.018). (**C**) The lateralization of frontal fast spindles (Spindle^Lat^) was significantly associated the overall *d’* (*p* = 0.042). Excluding a potential outlier (white dot) removed the significance (*p* = 0.110). (**D**) The lateralization of frontal coupling distance (Coupling^Lat^) was significantly associated with overall emotional difference, i.e., neutral *d’* – emotional *d’*, across all retrievals (*p* = 0.005). (**E**) Spindle^Lat^ was significantly associated with emotional difference in the 24-h retrieval (*p* < 0.001)

We first inspected the distribution of SO-coupled sleep spindles. Both frontal and central SO-coupled fast sleep spindles were nonuniformly distributed across the SO cycle (Rayleigh’s test *p* < 0.001). The grand mean degrees were −2.3° for frontal and 12.9° for central SO-spindles. Figure 3A displays the polar histograms of fast SO-spindles.

Second, we examined whether the oscillatory variables (spindle density, coupling distance, and REM theta), averaged between hemispheres, associated with recognition scores and their interaction with time and emotion. No main effects were found (Model 1 *p* values ≥ 0.743; Model 2 *p* values ≥ 0.489); that is, none of the variables associated with overall *d’*. Interaction with time was significant regarding central spindle density in Model 1 only [F(2, 52) = 4.024, *p* = 0.024, η_p_^2^ = 0.134; Model 2 F(2, 24) = 1.962, *p* = 0.151], and follow-up tests in Model 1 showed that central spindle density was significantly associated with *d’* retention between 12-h and 24-h measurements (i.e., 12-h *d’* – 24-h *d’*; t = 2.538, *p* = 0.018; Figure 3B) but not between immediate and 12-h measurements (t = −1.173, *p* = 0.251). Other oscillatory variables did not significantly interact with time (Model 1 *p* values ≥ 0.131; Model 2 *p* values ≥ 0.164). There were no significant interactions regarding emotion (Model 1 *p* values ≥ 0.307; Model 2 *p* values ≥ 0.398), nor were there three-way interactions between oscillatory variables and “time x emotion” (Model 1 *p* values ≥ 0.605; Model 2 *p* values ≥ 0.567). See Table 2 for Model 1 results and Supplementary material 2 for Model 2 results. Recollection-based analyses are shown in Supplementary material 1.

**Table 2:** Sleep oscillations and discriminability *d’* in Model 1

	Main effect		X time		X emotion		X time X emotion	
	F	*p*	F	*p*	F	*p*	F	*p*
Spindle density, frontal	0.110	0.743	1.691	0.194	0.357	0.556	0.507	0.605
Spindle density, central	0.019	0.892	4.017	0.024^*✝^	0.003	0.956	0.302	0.740
Coupling distance, frontal	0.095	0.760	0.831	0.441	1.086	0.307	0.195	0.823
Coupling distance, central	0.028	0.868	2.109	0.131	0.812	0.376	0.190	0.827
REM Theta	0.045	0.834	0.414	0.664	0.470	0.499	0.198	0.821
Spindle^Lat^	4.555	0.042*✝	0.426	0.655	3.002	0.095	5.792	0.005^**^
Coupling^Lat^	0.122	0.729	1.608	0.210	9.273	0.005^**^	0.733	0.485
Theta^Lat^	0.030	0.864	0.087	0.917	0.632	0.434	1.785	0.177

X time = the interaction between time and the oscillatory variable; X emotion = the interaction between emotion and the oscillatory variable. X time X emotion = three-way interaction between time, emotion, and the oscillatory variable. Covariates: sex, sleep duration, location. **p < 0.01; *p < 0.05. ^✝^Not significant after controlling for BDI and GAD-7 scores

### Oscillatory lateralization, discriminability, and emotional prioritization

Next, we investigated the lateralization variables and especially their interaction with emotion. Spindle^Lat^ showed a significant main effect on *d’* in Model 1 [F(1, 27) = 4.555, *p* = 0.042, ηp^2^ = 0.144; Figure 3C] but not when BDI and GAD-7 scores were controlled [Model 2 F(1, 25) = 1.188, *p* = 0.286, ηp^2^ = 0.045]. The scatterplot in Figure 3C shows a deviating value with overall *d’* of −2.75 SD. Excluding that participant made the association nonsignificant (t = 1.655, *p* = 0.110).

Neither Coupling^Lat^ nor Theta^Lat^ significantly predicted overall *d’* in Model 1 or 2 (*p* values ≥ 0.729). None of the lateralization variables interacted with time in either model (*p* values ≥ 0.210), but Coupling^Lat^ significantly interacted with emotion [Model 1 F(1, 27) = 9.273, *p* = 0.005, ηp^2^ = 0.256; Model 2 F(1, 25) = 7.572, *p* = 0.011, ηp^2^ = 0.232]. Follow-up regression test showed that Coupling^Lat^ associated negatively with the difference between neutral and emotional *d’* [Model 1 t = −3.045, *p* = 0.005; Model 2 t = −2.752, *p* = 0.011; Figure 3D). No interaction with emotion was found regarding Spindle^Lat^ or Theta^Lat^ (*p* values ≥ 0.095). Three-way interaction “Spindle^Lat^ x time x emotion” was significant [Model 1 F(2, 54) = 5.792, *p* = 0.005 , ηp^2^ = 0.177; Model 2 F(2, 50) = 5.119, *p* = 0.009, ηp^2^ = 0.170], denoting that the association between Spindle^Lat^ and emotional difference (neutral *d’* – emotional *d’*) varied across measurements. Examining how Spindle^Lat^ related to emotional difference in the three retrievals showed a significant association in 24-h retrieval (Model 1 t = 3.983, *p* < 0.001; Model 2 t = 3.602, *p* = 0.001) but not in immediate or 12-h retrieval (*p* values ≥ 0.177). See Figure 3E for illustration. No other three-way interactions were found (*p* values ≥ 0.168). See Table 2 for Model 1 results and Supplementary material 2 for Model 2 results. See Supplementary material 3 for *d’* in subgroups created by median split on Coupling^Lat^ and Spindle^Lat^. Recollection-based *d’* analyses are shown in Supplementary material 1.

Finally, we examined whether including handedness as a covariate affects the significant findings regarding Spindle^Lat^ and Coupling^Lat^. The main effect of Spindle^Lat^ on overall *d’* became nonsignificant [F(1, 24) = 3.108, *p* = 0.091, ηp^2^ = 0.115], whereas its interaction with “time x emotion” remained significant despite a decrease in effect size [F(2, 48) = 3.849, p = 0.028, ηp^2^ = 0.138]. An increase in effect size was seen regarding “Coupling^Lat^ x time” interaction [F(1, 24) = 11.423, *p* = 0.002, ηp^2^ = 0.322].

### Hit rate and false-alarm rate

We conducted a set of post-hoc analyses to explore the dynamics of hit rate and false alarm rate in those analyses that were found significant concerning recognition discriminability (*d’*). These included the repeated measures ANOVA on *d’* across immediate, 12-h, and 24-h measurements, as well as the significant follow-up analyses between *d’* and oscillatory variables: 1) central spindle density and 12-h to 24-h retention; 2) Spindle^Lat^ and emotional difference at 24-h retrieval; and 3) Coupling^Lat^ and overall emotional difference. From these analyses, we excluded the main effect of Spindle^Lat^ on overall *d’*, as we found that the association was heavily drawn by one value (Figure 3C). See also Supplementary material 3, which suggests that specifically 24-h emotional difference contributed to the association.

**Fig. 4:**
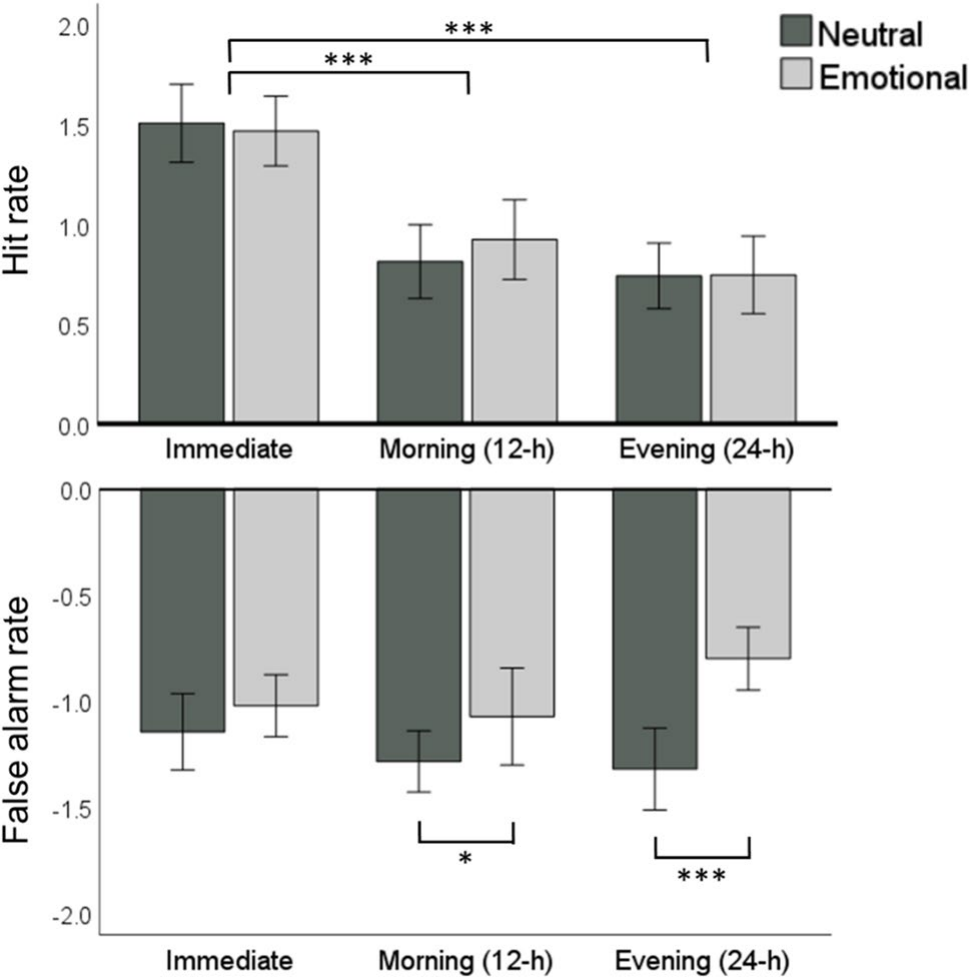
Hit rates and false-alarm rates for neutral and emotional pictures across immediate, 12-h, and 24-h retrievals. ***p < 0.001; *p < 0.05. Error bars indicate 95% confidence interval

Repeated measures ANOVA on neutral and negative hit rate across immediate, 12-h, and 24-h measurements showed a significant effect regarding time [F(2, 64) = 80.948, *p* < 0.001] but not emotion [F(1, 32) = 0.589, *p* = 0.448] or “time x emotion” [F(2, 64) = 0.804, *p* = 0.452]. Bonferroni-corrected, follow-up, pairwise comparisons showed that the immediate hit rate was higher than either 12-h (*p* < 0.001) and 24-h hit rate (*p* < 0.001), whereas no difference was found between 12-h and 24-h measures (*p* = 0.220). See Figure 4 for illustration.

A similar investigation of false-alarm rate revealed a significant effect of emotion [F(1, 32) = 26.388, *p* < 0.001] but not time [F(2, 64) = 1.551, *p* = 0.220]. The interaction “time x emotion” was significant [F(2, 64) = 9.220, *p* < 0.001]. Bonferroni-corrected, follow-up examination showed that the false-alarm rate was lower in neutral than emotional pictures overall and in 12-h (*p* = 0.039) and 24-h retrievals (*p* < 0.001). Also, the false-alarm rate for emotional items was higher in 24-h than in immediate (*p* = 0.019) or 12-h (*p* = 0.020) measurement, whereas no differences were found regarding neutral false-alarm rates (*p* ≥ 0.116). See Figure 4 for illustration.

Next, we re-ran the follow-up regression analyses with either hit rate or false-alarm rate as the dependent variable (instead of *d’*). Note that higher (positive) t value in hit rate indicates accurate recognition of target items, whereas lower (negative) t value in false-alarm rate indicates accurate rejection of foil items. Central spindle density was associated with 12-h to 24-h difference in hit rate (t = 2.541, *p* = 0.018) but not false-alarm rate (t = −0.426, *p* = 0.674). Spindle^Lat^ was significantly associated with 24-h emotional difference (neutral – emotional) regarding both hit rate (t = 2.396, *p* = 0.024) and false-alarm rate (t = −2.633, *p* = 0.014). Coupling^Lat^ was significantly associated with overall emotional difference in hit rate (t = −2.226, *p* = 0.035). The association regarding false-alarm rate displayed a nonsignificant trend (t = 1.783, *p* = 0.086).

## Discussion

This present study investigated the retention of neutral and emotional (negative) pictures over a delay comprising a full night of sleep. Memory retrievals were administered immediately after encoding and after 12-h and 24-h delays. The average recognition discriminability (*d’*) decreased over the retrieval occasions, whereas the 12-h to 24-h delay (spent awake) saw a pronounced decrease regarding emotional pictures, relative to neutral ones. The lateralization (right-to-left difference) of SO-spindle coupling distance was associated with overall neutral picture benefit, whereas lateralized sleep spindle density predicted the increase of neutral benefit between the 12-h and 24-h retrievals. Within this period, memory retention was predicted by central sleep spindle density.

Our primary goal was to examine how oscillatory lateralization during sleep associated with emotional prioritization in delayed picture recognition. We expected right-lateralized sleep oscillations to predict emotional memory benefit, in line with previous studies regarding REM theta (Nishida et al., 2009; Sopp et al., 2017). Instead, we found that right-lateralized NREM oscillations were associated with a neutral over emotional advantage in discriminability. First, spindle density lateralization predicted emotional discriminability difference at the 24-h retrieval. The current understanding of lateralized sleep spindles and their role in memory prioritization is deficient, because no existing research has examined memory and right-to-left contrast in spindle activity. Previous studies probing how nonlateralized (i.e., one electrode) frontal spindle activity in different hemispheres associates with emotional or neutral memory consolidation have provided inconsistent results. Post-sleep memory for neutral material has been linked to either left (Sopp et al., 2017) or right (Sopp et al., 2018) frontal spindles. Yet another study reported right frontal spindles to support the retention of emotional contexts (Cairney et al., 2014). However, the hemispheric differences in spindle-memory associations were minor in these studies, and thus no conclusions between spindle lateralization and selective off-line consolidation can be drawn based on these previous studies.

Critically, the associations between oscillatory lateralization and neutral memory benefit may not stem (only) from a bias in offline consolidation. We also found that the lateralization of mean SO-spindle coupling distance (i.e., relatively stronger “upstate” preference in the right hemisphere) predicted an overall emotional difference, such that an advantage for neutral items was observed already in the immediate retrieval prior to sleep. Indeed, it has been suggested that sleep spindles and their coupling with SOs reportedly mirror the properties of underlying brain networks (Gaudreault et al., 2018; Helfrich et al., 2018; Muehlroth et al., 2019). It may not be surprising that an asymmetry in these oscillations coincide with behavioral inclination, such as mnemonic prioritization, as observed in our study. Right hemisphere activity facilitates working memory, executive functioning, and visual recognition memory (Hilbert et al., 2019; Smirni et al., 2015; Spagna et al., 2020; Turriziani et al., 2008, 2012). Right dorsolateral prefrontal cortex is important for error awareness and conscious response monitoring (Harty et al., 2014). It is possible that individuals with right-lateralized spindle activity in the current study had more efficient neural network for tasks requiring conscious attention and rapid monitoring of response options, contributing to both encoding and retrieval. The effect may have been more emphasized for neutral pictures. As proposed by Sopp et al. (2017), neutral material engages strategic processing mechanisms during intentional memory encoding. Top-down control in such situation may dilute the effect of bottom-up, stimuli-driven emotional cues (Bennion et al., 2016). Regarding retrieval, right-lateralized spindles were associated not only with neutral-to-emotional benefit in correct recognitions (hit rate) but also correct rejections (false-alarm rate). This suggests a strategic and/or emotional response bias at retrieval, although better understanding requires additional research.

The key question is then the role of sleep-related memory consolidation in our study. We found that central fast spindle density predicted overall (regardless of emotion) memory retention in the 12-h to 24-h delay that was spent awake. The association was mostly drawn by hit rate, i.e., correct recognitions of target pictures, implying that spindle-related processes facilitated offline consolidation of encoded memories during sleep. This aligns well with previous behavioral and theoretical accounts regarding sleep spindles (Clemens et al., 2005; Goder et al., 2015; Halonen et al., 2019; Klinzing et al., 2019; Rosanova & Ulrich, 2005; Schabus et al., 2004). Moreover, some reports indeed argue that consolidation during sleep would stabilize memories against wake-time interference (Ellenbogen et al., 2009; Payne et al., 2012).

Pieced together, picture recognition performance in our study seems to have been influenced by the combined effects of (frontal) oscillatory lateralization and (central) spindle density: respectively, an inter-individually variable bias in initial encoding and response control, incrementally augmented by offline memory consolidation. It is not clear how exactly the roles of spindles and coupling differ, lateralized or not. Speculatively, SO-spindle distance, an average measure over several events, is more a reflection of neuroanatomical underpinnings, whereas spindle density captures the number of oscillatory events, and thus, may better predict mechanistic consolidation.

It remains insufficiently answered why the recognition discriminability was higher for neutral than emotional pictures. This finding is at odds with numerous studies that have observed better retrieval for emotional memories, relative to neutral, after sleep (Bennion et al., 2016; Halonen, Kuula, Lahti, et al., 2021b; Hu et al., 2006; Payne et al., 2008, 2012). For example, a study that ran a comparable paradigm to examine emotional versus neutral memory found that especially emotional material remained durably in memory for 24 hours in the group that slept right after the encoding (Payne et al., 2012). The authors presumed that sleep stabilized those memories that were tagged salient during encoding. Our examination on hit rate and false-alarm rate showed that the emotional difference at 24-h retrieval was driven by increased false recognitions of emotional pictures. This is in concordance with evidence indicating that negative, relative to neutral, material evokes false recognitions more easily (Ferré et al., 2019; Gallo et al., 2009). However, hit rates were rather unexpectedly (Ferré et al., 2019) equal between the emotional categories across the retrievals. Given that the preservation of hit rate represents memory retention (as discussed previously), it appears that neither neutral or emotional pictures were prioritized in offline consolidation by selective “tagging” (Kim & Payne, 2020). What might have equalized the saliency of pictures with different emotional charge?

Methodological choices should be considered. Emotional memory benefit is typically seen in paradigms where neutral and emotional material are intermixed during encoding such that they compete for the same processing resources (Cairney et al., 2014; Hu et al., 2006; Nishida et al., 2009; Payne et al., 2012; Sopp et al., 2017). When neutral and emotional materials alternate rapidly and/or even overlap, bottom-up attentional processing may prioritize the encoding and subsequent consolidation of emotional objects, while leading to the suppression of less salient (neutral) material (Cairney et al., 2014). A recent review concludes that the majority of studies examining such emotion trade-off paradigms report specific memory benefits for emotional material (Davidson et al., 2021). In the memory task of the present study, the stimuli were not intermixed but presented in two sets, each set consisting of one emotion; the participants first encoded a set containing only neutral stimuli, and then a set containing only emotional stimuli. This type of presentation potentially induced habituation, prevented competition between emotional and neutral stimuli, and diluted the *distinctiveness* of emotional material at encoding, a property necessary for memory benefit (Hourihan et al., 2017). It also is possible that such stimulus presentation negated a potential emotional benefit by introducing a primacy advantage for neutral pictures that were presented first, whereas increasing inattention or fatigue may have impaired encoding during the subsequent emotional picture set.

The discussion above possibly accounts for the lack of associations regarding REM theta in our study. Previously, lateralized REM theta has been associated with emotional memory consolidation in humans (Nishida et al., 2009; Sopp et al., 2017), endorsing its role as the major oscillatory range for emotional processing. Assuming that the arrangement of our memory task mitigated the effect of emotion on memory processing, the contribution of (lateralized) REM theta may have become negligible. What remains an open question is whether theta lateralization in REM sleep is the sole marker for emotional offline processing. The studies by Nishida et al. (2009) and Sopp et al. (2017) did not investigate the hemispheric asymmetry of NREM oscillations. Thus, studies using memory paradigms that are known to yield emotion-related benefits are warranted to further chart the topic.

Finally, the questionnaire scores for depression and anxiety symptomology were negatively associated with discriminability, especially in the 24-h retrieval, central spindle density, and spindle density lateralization. It thus appears that prevalent affective constitution intertwines with overnight memory consolidation and the related oscillatory characteristics. Regarding spindle lateralization, we found relatively lower right-hemisphere spindle activity in those with high anxiety scores. Given the activity-dependent modulation of local spindle activity (Morin et al., 2008; Yordanova, Kolev, et al., 2017b), this finding seems to be in contrast to the reportedly right-lateralized brain activity in anxious and depressed individuals (Bruder et al., 1997, 1999, 2017; Moratti et al., 2008). A closer look at the literature renders the hemispheric asymmetry less clear-cut. For example, differential lateralization patterns have been observed depending on anxiety subtype (anxied arousal vs. worry) and whether depression and anxiety are comorbid (Bruder et al., 2017; Heller & Nitschke, 1998). In a study on working memory, anxiety was found to be associated with bilateral processing, relative to healthy controls who relied on stronger right-lateralized processing (Balderston et al., 2020). The participants in the present study did not represent any population with diagnosed disorders, and thus, the oscillatory characteristics in our data are hardly generalizable to major affective conditions. Of note, BDI and GAD-7 scores appeared partially distinct from oscillatory lateralization in terms of memory bias; controlling for the questionnaire scores did not dilute the statistical significance of spindle/coupling lateralization on neutral memory preference. Further research is necessary to develop a better understanding on how mood and anxiety relate to the lateralization of sleep oscillations and if this lateralization manifests differently in subclinical and clinical populations.

### Strengths and limitations

This study has clear strengths. First, memory retrieval was measured three times: immediately after the encoding, in the morning after a full night of sleep, and finally the next evening. Commonly, performance is measured immediately after encoding and right after a sleep period, which precludes observing how robustly memories survive the wake-related interference. Second, instead of focusing on a single oscillatory variable, we included oscillations that characterize both REM and NREM sleep. This adds to knowledge regarding the relative importance of sleep oscillatory events for emotional memory performance—a field previously focused on REM theta. Third, investigating hit rates and false-alarm rates clarified how different processes contributed to the discrimination between targets and foils over time and in relation to sleep oscillations. Fourth, collecting data on anxiety and depression symptomology enabled us to address their role in the observed phenomena.

There are limitations that should be addressed. First, the sample size was rather small, compromising reliability. However, we selected our analytical approach carefully to avoid spurious associations. Second, as a strategic choice we did not mix neutral and negative stimuli during encoding. Because the stimuli sets represented a single emotion, the participants could have developed response tendencies specific for only neutral or emotional stimuli, thus obviating a possible benefit of emotional competition as discussed previously. Additionally, not counterbalancing the presentation order for neutral and emotional picture sets may have introduced confounding effects by primacy and fatigue. Third, the study did not include a wake-first condition. To understand the phenomenological nature of the observed neutral memory benefit more deeply, it would have been valuable to include participants who instead of sleeping would have remained awake after the encoding. This remains an aspect to be explored in future studies. Finally, assessment of the effect of handedness was lacking due to relatively many missing values, precluding a proper investigation of the issue.

## Conclusions

This study found a neutral over emotional benefit in picture recognition discriminability at retrieval 24 hours after encoding. Sleep spindles associated with memory retention in general, whereas oscillatory lateralization (right-to-left difference) during NREM sleep predicted the recognition advantage of neutral over emotional memories. Sleep oscillations do not only facilitate offline consolidation, but their lateralization properties may mirror individual neuroanatomy. This may influence how memory processes also take place during wakefulness. Our findings contribute to an unexplored area in sleep-related memory research. Through studying oscillatory lateralization, we provide novel perspectives on how the memory for neutral and emotional information relates to individual factors. However, further research with diverse memory paradigms is required.

## Supplementary Information

ESM 1 (DOCX 513 kb)

## Data Availability

None of the data or materials for the experiments reported are available, and none of the experiments were preregistered.
