# Supplementary material for: Right-lateralized sleep spindles are associated with neutral over emotional bias in picture recognition: An overnight study
Source: Cogn Affect Behav Neurosci. 2023 Jun 12;23(5):1445–59. doi: 10.3758/s13415-023-01113-4 (PMC10260275; doi:10.3758/s13415-023-01113-4)
Supplement: Supplementary file 1 — (DOCX 513 kb) [file 13415_2023_1113_MOESM1_ESM.docx]

**Supplementary material 1: recollection**

We conducted an analysis where only recollection responses were included in calculating recognition discriminability (*d’*). Here, *d’* differed statistically significantly between retrieval occasions [*F*(2, 62)= 95.955, p < 0.001]. Also, emotion had a statistically significant main effect in the model [*F*(1, 31) = 13.084, *p* = 0.001]. Finally, time and emotion had a statistically significant interaction in the model [*F*(2, 62) = 14.117, *p* < 0.001]. Post hoc analyses conducted using Bonferroni corrected pairwise comparisons revealed that *d’* decreased from immediate retrieval (*M* = 2.84, *SE* = 0.10, p < 0.001) to 12-h retrieval (*M* = 2.24, *SE* = 0.11, p < 0.001). Also, the decrease in *d’* from immediate to evening retrieval (*M* = 1.87, *SE* = 0.11) was statistically significant (p < 0.001). Likewise, the decrease in *d’* from 12-h retrieval to 24-h retrieval was statistically significant (p < 0.001). Additionally, *d’* for neutral items at 24-h retrieval (*M* = 2.19, *SE* = 0.13) was statistically significantly (*p* < 0.001) greater than *d’* for emotional items (*M* = 1.54, *SE* = 0.11). Figure S2 displays the results of the two-way repeated measures ANOVA described above.


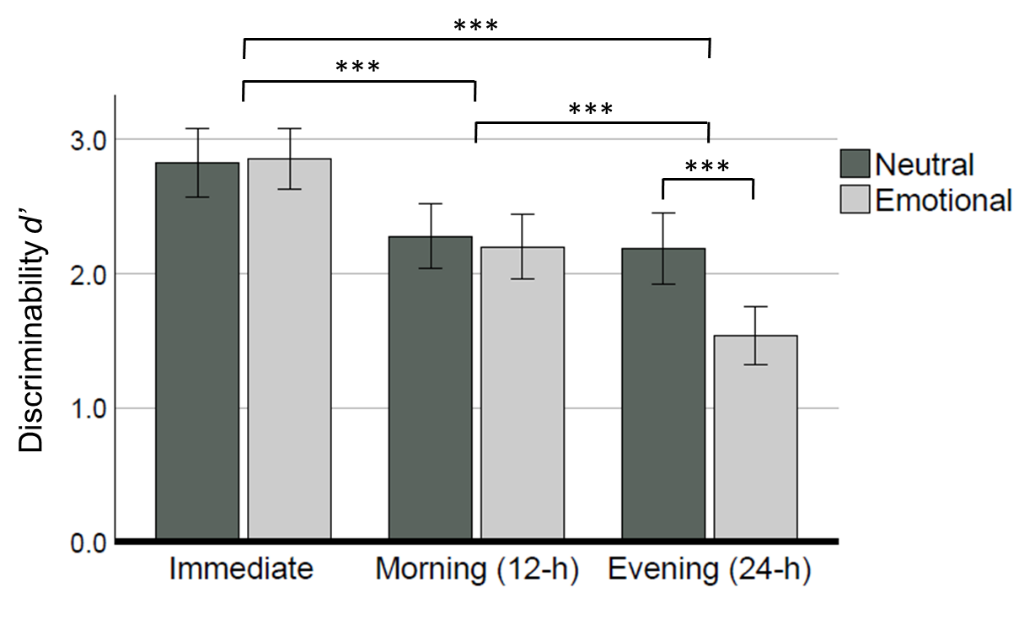


**Figure S1.** The development of recollection-based discriminability (d’) for neutral and emotional items across the time points when memory retrieval tasks were performed. *** p < 0.001. Error bars indicate 95% confidence interval.

Examining the main effects and interactions (with time, emotion and ‘time x emotion’) between oscillatory/lateralization variables and recollection-based discriminability *d’* revealed significant interactions between Spindle^Lat^ and time [Model 1 F(2,54) = 3.444, p = 0.039; Model 2 F(2, 50) = 2.411, p = 0.100], and Spindle^Lat^ and emotion [Model 1 F(1, 27) = 5.688, p = 0.024; Model 2 F(1, 26) = 2.567, p = 0.122], and Coupling^Lat^ and emotion [Model 1 F(1, 27) = 5.582, p = 0.026; Model 2 F(1, 26) = 4.281, p = 0.049]. The coefficients and p values for Model 1 and Model 2 are shown in Tables S1 and S2, respectively.

| Table S1. Sleep oscillations and recollection-based discriminability *d’* in Model 1. | | | | | | | | | |
| --- | --- | --- | --- | --- | --- | --- | --- | --- | --- |
|  | Main effect | | X time | | X emotion | | X time X emotion | | |
|  | F | p | F | p | F | p | | F | p |
| Spindle density, frontal | 0.218 | 0.645 | 1.145 | 0.326 | 0.334 | 0.568 | | 0.648 | 0.527 |
| Spindle density, central | 0.018 | 0.895 | 1.859 | 0.166 | 2.227 | 0.148 | | 1.925 | 0.156 |
| Coupling distance, frontal | 0.241 | 0.627 | 0.088 | 0.916 | 0.284 | 0.599 | | 0.784 | 0.462 |
| Coupling distance, central | 0.000 | 0.983 | 1.384 | 0.259 | 0.340 | 0.564 | | 1.863 | 0.165 |
| REM Theta | 0.442 | 0.512 | 0.516 | 0.600 | 0.012 | 0.915 | | 0.249 | 0.780 |
| Spindle^Lat^ | 3.622 | 0.068 | 3.444 | 0.039* | 5.688 | 0.024* | | 1.634 | 0.205 |
| Coupling^Lat^ | 0.110 | 0.743 | 0.943 | 0.396 | 5.582 | 0.026* | | 0.869 | 0.425 |
| Theta^Lat^ | 0.450 | 0.508 | 0.072 | 0.931 | 0.215 | 0.647 | | 0.014 | 0.986 |
| X time: the interaction between time and the oscillatory variable. X emotion: the interaction between emotion and the oscillatory variable. X time X emotion: three-way interaction between time, emotion and the oscillatory variable. Covariates: sex, sleep duration. * p < .05. | | | | | | | | | |

| Table S2. Sleep oscillations and recollection-based discriminability *d’* in Model 2. | | | | | | | | | |
| --- | --- | --- | --- | --- | --- | --- | --- | --- | --- |
|  | Main effect | | X time | | X emotion | | X time X emotion | | |
|  | F | p | F | p | F | p | | F | p |
| Spindle density, frontal | 0.595 | 0.448 | 1.124 | 0.333 | 0.075 | 0.786 | | 0.535 | 0.589 |
| Spindle density, central | 0.306 | 0.585 | 0.834 | 0.440 | 1.141 | 0.296 | | 1.371 | 0.264 |
| Coupling distance, frontal | 0.020 | 0.887 | 0.130 | 0.878 | 0.005 | 0.946 | | 0.694 | 0.504 |
| Coupling distance, central | 0.055 | 0.816 | 1.315 | 0.278 | 0.027 | 0.870 | | 1.606 | 0.211 |
| REM Theta | 1.002 | 0.326 | 0.831 | 0.442 | 0.090 | 0.767 | | 0.344 | 0.710 |
| Spindle^Lat^ | 1.188 | 0.286 | 2.411 | 0.100 | 2.567 | 0.122 | | 1.142 | 0.327 |
| Coupling^Lat^ | 0.003 | 0.953 | 0.792 | 0.458 | 4.281 | 0.049* | | 0.960 | 0.390 |
| Theta^Lat^ | 0.742 | 0.397 | 0.026 | 0.975 | 0.338 | 0.566 | | 0.125 | 0.882 |
| X time: the interaction between time and the oscillatory variable. X emotion: the interaction between emotion and the oscillatory variable. X time X emotion: three-way interaction between time, emotion and the oscillatory variable. Covariates: sex, sleep duration. * p < .05. | | | | | | | | | |

Follow-up tests in Model1 revealed that Spindle^Lat^ was positively associated with retention during 12-h to 24-h delay (t = 2.366, p = 0.026) (Figure S2A) but not between immediate and 12-h delay (t = 0.558, p = 0.582). Both Spindle^Lat^ and Coupling^Lat^ were associated with overall emotional difference (t = 2.385, p = 0.024 and t = -2.363, p = 0.026) (Figures S2B and S2C, respectively).


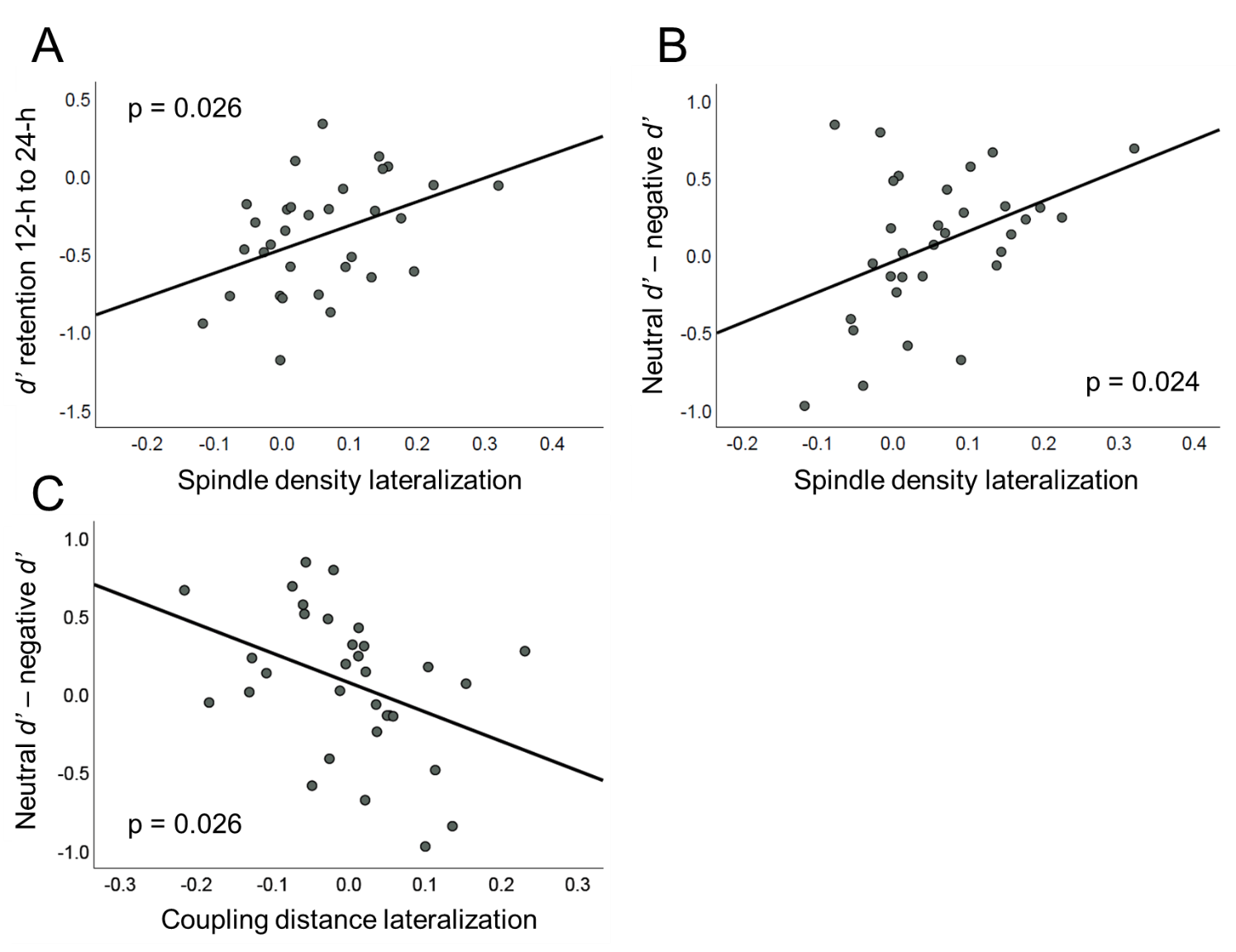


Figure S2. Lateralization and overall emotional difference in recollection-based recognition. (A) Spindle density lateralization was associated with retention performance between 12-h and 24-h retrievals (p = 0.026) and (B) with emotional difference across all retrievals (p = 0.024). Coupling distance lateralization was associated with emotional difference (p = 0.026) (C).

**Supplementary material 2: Repeated measures ANCOVA in Model 2**

| Table S3. Sleep oscillations and discriminability *d’* in Model 2. | | | | | | | | | |
| --- | --- | --- | --- | --- | --- | --- | --- | --- | --- |
|  | Main effect | | X time | | X emotion | | X time X emotion | | |
|  | F | p | F | p | F | p | | F | p |
| Spindle density, frontal | 0.493 | 0.489 | 1.360 | 0.266 | 0.739 | 0.398 | | 0.574 | 0.567 |
| Spindle density, central | 0.494 | 0.489 | 2.362 | 0.105 | 0.174 | 0.680 | | 0.255 | 0.776 |
| Coupling distance, frontal | 0.007 | 0.934 | 0.746 | 0.479 | 0.341 | 0.564 | | 0.120 | 0.887 |
| Coupling distance, central | 0.152 | 0.699 | 1.874 | 0.164 | 0.307 | 0.585 | | 0.077 | 0.926 |
| REM Theta | 0.411 | 0.527 | 0.834 | 0.440 | 0.649 | 0.428 | | 0.472 | 0.626 |
| Spindle^Lat^ | 1.188 | 0.286 | 0.127 | 0.881 | 1.458 | 0.239 | | 5.119 | 0.009** |
| Coupling^Lat^ | 0.001 | 0.971 | 1.410 | 0.254 | 7.572 | 0.011* | | 0.515 | 0.600 |
| Theta^Lat^ | 0.002 | 0.965 | 0.226 | 0.799 | 0.915 | 0.348 | | 0.985 | 0.380 |
| X time = the interaction between time and the oscillatory variable; X emotion = the interaction between emotion and the oscillatory variable. X time X emotion = three-way interaction between time, emotion and the oscillatory variable. Covariates: sex, sleep duration, location, BDI and GAD-7 scores. ** p < .01 * p < .05. | | | | | | | | | |

**Supplementary material 3: low vs. high lateralization**

We performed a supplementary analysis to further illustrate the impact of Coupling^Lat^ and Spindle^Lat^ on *d’* across the three retrievals (immediate, 12-h and 24-h). First, we created two subgroups by median split on Coupling^Lat^. One-way ANOVA between the subgroups showed a significant difference regarding central spindle density (p = 0.024), such that those in the low Coupling^Lat^ had higher density (4.01 vs. 3.56). No other differences in sample characteristics or oscillatory measures were found (p values ≥ 0.171).

A mixed ANCOVA (3 retrievals, 2 emotions; Model 1) was rerun with Coupling^Lat^ group introduced as a two-level between-subjects factor. The main effect of Coupling^Lat^ group on overall *d’* was not statistically significant [F(1, 27) = 0.076, p = 0.784] nor did it interact with time [F(2, 54) = 1.501, p = 0.232]. The interaction between Coupling^Lat^ group and emotion was significant [F(1, 27) = 12.811, p = 0.001], whereas its three-way interaction with ‘time x emotion’ was not [F(2, 54) = 0.869, p = 0.425]. Follow-up examination showed that in the low Coupling^Lat^ group, the averaged neutral *d’* was higher than the averaged negative *d’* (2.42 vs. 1.95; p < 0.001) whereas no difference was found in the high Coupling^Lat^ group (p = 0.679). Within-group pairwise comparisons between neutral and emotional accuracy scores in the retrieval occasions showed that in the low Coupling^Lat^ group, neutral *d’* was higher than emotional *d’* in immediate and 24-h retrieval (Bonferroni-corrected p = 0.030 and p = 0.003, respectively). The Figure S3A illustrates the *d’* scores across the retrieval occasions in both Coupling^Lat^ groups.

For the next analysis, the participants were divided into two groups by median split on Spindle^Lat^. The Spindle^Lat^ subgroups did not differ in any of the sample characteristics or oscillatory measures presented in Table 1 (p-values ≥ .092). Aligning with the correlation analysis between Spindle^Lat^ and questionnaire scores, the two groups differed in GAD-7 (p = 0.009).

In the mixed ANCOVA (Model 1) with Spindle^Lat^ as a between-subjects factor, the main effect of Spindle^Lat^ group on overall *d’* was not statistically significant [F(1, 27) = 1.417, p = 0.244] nor did it interact with time [F(2, 54) = 1.384, p = 0.259] or emotion [F(1, 28) = 2.109, p = 0.158]. However, there was a statistically significant interaction between time, emotion and Spindle^Lat^ group [F(2, 54) = 3.576, p = 0.035], indicating differential development of *d’* for neutral and emotional material in the two Spindle^Lat^ groups as a function of time. Bonferroni-corrected follow-up pairwise comparisons revealed that only in the high Spindle^Lat^ group, neutral *d’* was significantly greater than emotional *d’* in the 24-h retrieval (p = 0.001). Between the subgroups, only neutral *d’* in the 24-h retrieval was significantly different (p = 0.018). Figure S5 illustrates the results of the mixed ANOVA analyses.


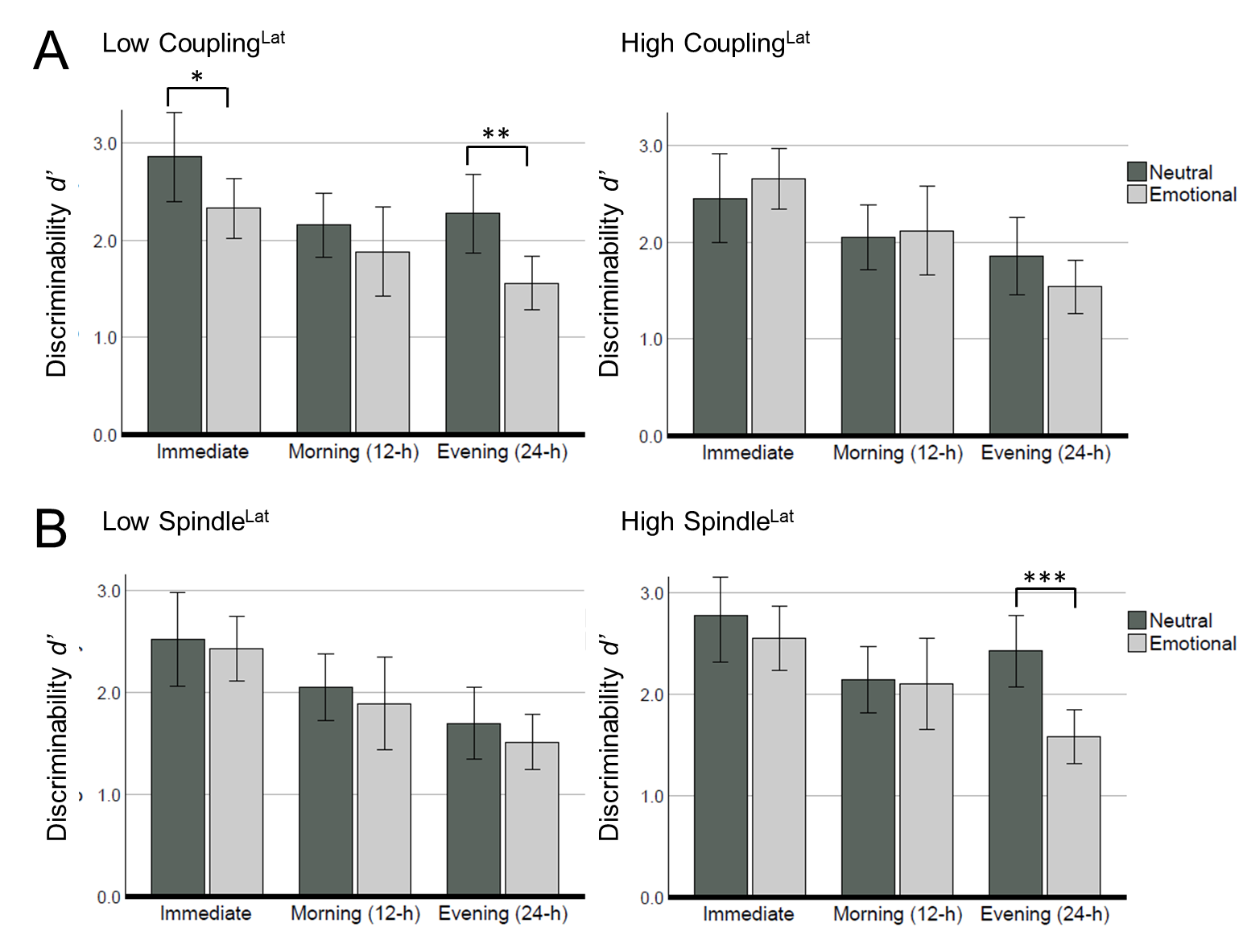


Figure S3. Discriminability *d’* across memory retrievals in subgroups by median split on oscillatory lateralization. The graphs display *d’* scores for Low and High Coupling^Lat^ (A) and Spindle^Lat^ (B) subgroups. Error bars indicate 95% confidence interval. *** p < 0.001; ** p < 0.01; * p < 0.05.
